# Characterization of an Innovative Detector Based on Scintillating Fiber for Personalized Computed Tomography Dosimetry

**DOI:** 10.3390/s22010090

**Published:** 2021-12-23

**Authors:** Clément Devic, Johann Plagnard, Mélodie Munier

**Affiliations:** 1 Fibermetrix, 67960 Entzheim, France; clement.devic@fibermetrix.fr; 2 CEA, List, Laboratoire National Henri Becquerel (LNE-LNHB), Université Paris-Saclay, 91120 Palaiseau, France; johann.plagnard@cea.fr

**Keywords:** dosimetry, plastic scintillator, fiber optic sensor, computed tomography, X-ray, light collection, radiation monitoring, diagnostic radiology

## Abstract

For technical and radioprotection reasons, it has become essential to develop new dosimetric tools adapted to the specificities of computed tomography (CT) to ensure precise and efficient dosimetry since the current standards are not suitable for clinical use and for new CT technological evolution. Thanks to its many advantages, plastic scintillating fibers (PSF) is a good candidate for more accurate and personalized real-time dosimetry in computed tomography, and the company Fibermetrix has developed a new device named IVISCAN^®^ based on this technology. In this study, we evaluated performances of IVISCAN^®^ and associated uncertainties in terms of dose-rate dependence, angular dependence, stability with cumulative dose, repeatability, energy dependence, length dependence, and special uniformity in reference and clinical computed tomography beam qualities. For repeatability, the standard deviation is less than 0.039%, and the absolute uncertainty of repeatability lies between 0.017% and 0.025%. The deviation between IVISCAN^®^ and the reference regarding energy dependence is less than 1.88% in clinical use. Dose rate dependence results show a maximum deviation under ±2%. Angular dependence standard deviation *σ* is 0.8%, and the absolute uncertainty was 1.6%. We observed 1% of variation every 50 Gy steps up to a cumulative dose of 500 Gy. Probe response was found to be independent of the PSF length with a maximum deviation $\Delta D_{size}$ < 2.7% between the IVISCAN^®^ probe and the 1 cm PSF probe. The presented results demonstrated that IVISCAN^®^ performances are in accordance with metrology references and the international standard IEC61674 relative to dosemeters used in X-ray diagnostic imaging and then make it an ideal candidate for real-time dosimetry in CT applications.

## 1. Introduction

Computed Tomography (CT) is a non-invasive, rapid, and extremely accurate diagnostic tool for physicians and has had an undeniable impact on healthcare. CT’s technological improvements have allowed a wide clinical use, and the number of procedures performed has steadily increased over the past decades [1]. It has thus increased by 20% in the United States between 2006 and 2016, by 58% in France between 2002 and 2017, and by 40% between 2007 and 2016 in Germany [2,3,4,5]. The same trend is observed in countries with a comparable healthcare system [6]. This increase in procedures is also accompanied by an increase in the average annual effective dose received by the population in diagnostic radiology, which now stands at 74.2% in France, 63% in the United States, and 67% in Germany [3,5]. The issue of the impact of low-dose radiation on health has been raised, not only in terms of radiation-induced cancers [7,8] but also in terms of non-cancerous effects such as cataracts or cardiovascular diseases [9], leading to the need for dose uncertainty in CT to be as small as possible [6]. However, these studies have limitations, particularly in terms of dosimetry [10]. In addition, a large number of patients undergo multiple examinations and are sometimes exposed to an effective dose higher than the 100mSv threshold often considered as a significant stochastic risk [11,12,13]. A 40% increase in patients who received an effective dose of more than 100 mSv was observed in France between 2012–2014 and 2015–2017 [5]. Underestimated or overestimated patient dosimetry leads to wrong priorities for medical radiation protection research and incorrect dose management of these patients who do not have an adequate perception of the risks linked to ionizing radiation [14]. Dosimetry is also crucial for pregnant patients because specific dose measurements and accurate estimation of the dose to the embryo/fetus may be required to be above 10 mGy, and abortion may be considered if the dose is above 100 mGy [15]. Finally, high accuracy is also needed for dose measurements used to compare different procedures and for reliable regulatory controls [6]. Facing these questions, the dose metric used in CT, i.e., the Computed Tomography Dose Index (CTDI) and the Dose Length Product (DLP), suffer from limitations such as the traceability to primary quantities for CDTI and for both the representativity to the patient’s morphology in addition to the CT parameters [6].

**Figure 1:**
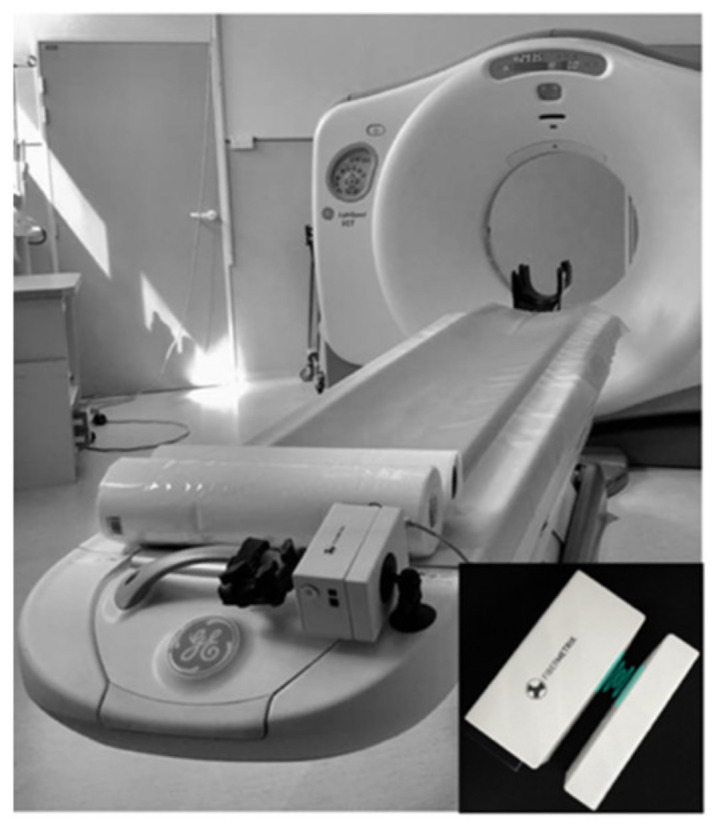
IVISCAN^®^ dosemeter installed on a CT scan. Illustration at the bottom right shows the photometer and the induction charging station.

Scintillation is a relatively old technique for detecting ionizing radiation [16], but the first modern scintillating fibers were suggested later [17,18,19]. Their development over the past few decades has made new dosimetry applications possible, particularly in the medical environment [20,21]. Scintillating optic fiber dosemeters (SFD) have also been validated to low-energy beam application in radiology. Since then, several developments have taken place without leading to a commercial product [20,22,23,24,25]. SFD system is beginning to be commercialized only for radiotherapy applications. As an example, the Exradin W1 and W2 Scintillator dosemeters based on this technology have been marketed for a few years by Standard Imaging Inc., Middleton, WI, USA [26,27,28]. Many advantages, such as compactness, high sensitivity, water-equivalence, radiolucency, real-time dose monitoring with high time resolution, and wide dynamic range of measured dose from a uGy to several Gy [21], make plastic scintillating fibers (PSF) a good candidate for more accurate and personalized real-time dosimetry in CT [21]. Moreover, in the radiodiagnostic energy range, SFDs are not disturbed by the Cherenkov, effect which is an interference that remains difficult to fully remove in radiotherapy applications and can causes significant measurement uncertainty. Moreover, passive material without electronic devices in the dosemeter probe head can cause harm to patient. Fibermetrix has designed the first SFD specific to CT imaging, the IVISCAN^®^, which is capable of measuring dose and dose-rate and evaluating CTDI and DLP in real-time during CT examinations. Finally, thanks to their wide detection length, it can overcome the limitations of reference dosimetry tool such as 100 mm pencil ionization chambers since the collimations on the latest generation of CT scans now exceed its detection length [6]. In clinical routine, the dosemeter probe is positioned all along the CT couch under the mattress where the patient takes place and automatically performs measurements. The photometer, which collects the light emitted by the probe, is positioned at the end of the CT-couch. A charging station, placed on the fixed part of the table, allows automatic induction charging of the system (Figure 1).

This paper aims to investigate the performance of this IVISCAN^®^ real-time dosimetry system in clinical use. In terms of metrological considerations, a detector should have favorable properties such as repeatability, dose, dose rate, and energy independence, minimal angular dependence, independence from prior radiation exposure, minimal radiation damage, and minimal temperature response. PSF temperature response has been studied substantially in the past and shows a small temperature dependence within the measured range. The measured temperature coefficient values vary from −0.03 to −0.15% per °C in a 10 to 30 °C range, depending on the study [29,30]. Moreover, in view of the conditions under which IVISCAN^®^ is used, the sensing probe is never in contact with the patient, so the probe remains at room temperature and will only vary by a few degrees at most because of the temperature control in the CT examination rooms. This component can be therefore neglected and will not be detailed in this work. Dose response linearity have also been studied in previous work and show a very linear response over the exposure range of interest in diagnostic radiology [28,31].

In this study, we evaluated repeatability, dose-rate dependence, stability with cumulative dose, angular dependence, spatial uniformity, and energy dependence in terms of air kerma and measurement uncertainties associated when used in clinical routine in CT imaging. Energy response of the IVISCAN^®^ system has already been evaluated in a previous work by simulation and comparison to standard detectors but never in clinical use [32]. We also estimated the effect of PSF length on the SFD response.

## 2. Materials and Methods

### 2.1. Dosimetry System

**Figure 2:**
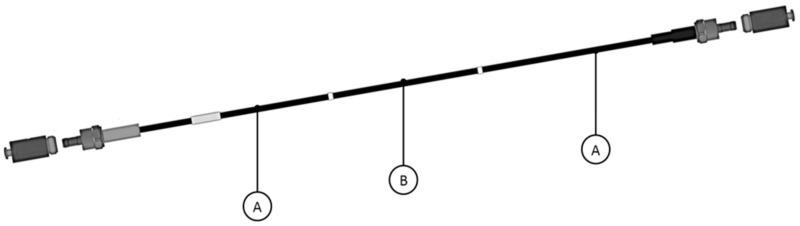
IVISCAN^®^ probe. Total length = 650 cm; sensing probe B length (delimited by the two white marks) = 200 cm. Lengths of the light guides A = 120 cm (left side) and 330 cm (right side). Each end has an SMA connector.

In this study, we tested the IVISCAN^®^ dosimetry system composed of an optical fiber probe connected to a two-channel photometer. Both devices from Fibermetrix^®^ company (Strasbourg, France). The probe consists of a BCF-12 (Saint Gobain, Courbevoie, France) PSF connected to two clear plastic optical fibers (POFs) at each end. The whole probe is surrounded by black Hytrel cladding. When placed in a radiation field, the BCF-12 emits scintillation photons with an emission peak at 435 nm. The emitted photons was then guided by the POFs and collected by two photomultiplier modules H10721-110 (Hamamatsu) that convert them to an electrical signal. Therefore, the system collects the light from the two side of the PSF and operates in photon counting mode. The PSF is 0.5 mm diameter by 200 cm long (0.39 cm^3^). The clear POFs are 0.5 mm diameter by approximately 120 cm and 330 cm long. The Hytrel sheath is 2.1 mm external diameter. Figure 2 shows a schematic drawing of the probe.

This specific design enables the probe to be placed in the CT couch, under the mattress, forming a U-shape at the head side of the couch, and the photometer to be placed to the end of the couch (foot side) where the patient and the examination is not impaired. In these conditions, the PSF part is positioned in-line along the z-axis allowing dose measurements all over the CT couch.

The resolution time of the IVISCAN^®^ dosemeter is 1 ms so as to visualize all the X-ray tube rotations in detail (full rotation time of a CT X-ray tube may be fast as 0.3 s). This type of data acquisition technique is then very useful for tube current modulation dosimetry studies.

### 2.2. Measurement Conditions

We investigate the dosimetric characteristics of IVISCAN^®^ for energies and filtration used for CT imaging. These characteristics were repeatability, energy dependence in air kerma, dose-rate response linearity (in air kerma rate), angular dependence, and stability with cumulative doses. We also evaluated the response deviation for different PSF lengths.

In order to achieve the most reliable repeatability, energy, and dose-rate dependence measurements, we needed stable and well-characterized radiation qualities. Considering this, we placed the IVISCAN^®^ dosemeter in the calibration reference conditions at the CEA LIST LNHB (Laboratoire National Henry Becquerel), which is an independent French primary laboratory for metrology of ionizing radiation. The references in terms of air kerma are obtained with a free-air ionization chamber, in the domain of low- and medium-energy X-ray dosimetry.

For this study, a large panel of radiation qualities was used so as to cover the entire radiology energy range. Table 1 gives the main specifications of the radiation quality, i.e., tube voltage, HVL, and effective energy. More detailed information can be found in ISO 4037, IEC61267, and BIPM(RI)I-K3. Most popular CT manufacturers give the 1st HVL values between 5 and 15 mm Al for tube voltage of 70 to 140 kV, corresponding to effective energies between 42 and 100 keV.

Due to the probe length, the calibration procedure had to be adapted so that the entire 200 cm sensing part of the probe was included in the irradiation beam. Consequently, the calibration procedure in air kerma is also described in this paper.

**Figure 3:**
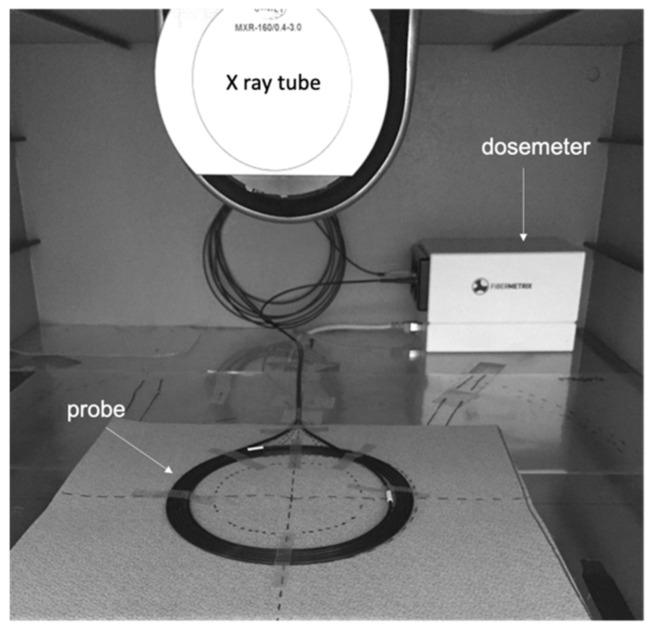
IVISCAN^®^ probe placement in the sealed irradiation facility.

**Figure 4:**
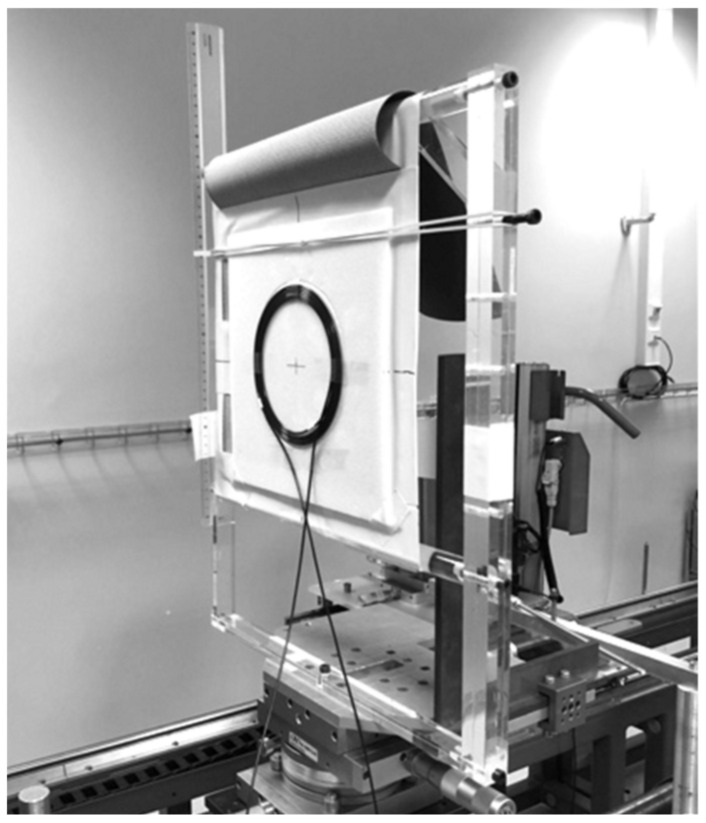
IVISCAN^®^ probe placement on the calibration bench at LNHB. Detector–source distance is 120 cm. The field was considered homogeneous within the polystyrene plate.

The validation of the energy dependence correction was performed under real use on a Siemens SOMATOM^®^ Definition Edge for all X-ray tube voltage/filtration pairs. The validation of the dose-rate and angular independence were performed also under real use on a Canon Aquilion ONE^TM^ Genesis. The remaining characterization measurements were performed with a Gulmay 160 kV generator and a Comet X-ray tube placed in a sealed irradiation chamber (Figure 3). The beam size is 19 cm diameter on the measurement plan. The placement of the IVISCAN^®^ probe was the same as for the calibration (Figure 4). Reference dose values for the validation of the energy dependence correction and the remaining characterization measurements were obtained with an Unfors Raysafe^TM^ X2 pencil ion chamber, which is the reference dosemeter for CT dose measurements. 

The beam stability was checked from reference dose measurements before and after the measurements were performed.

### 2.3. Calibration

We developed a novel calibration procedure for the irradiation measurements because the beam sizes in reference conditions were smaller than the 200 cm effective length of the probe. Thus, the probe is rolled on the whole length of the active part and placed on a expanded polystyrene support with 3 mm, thickness as presented on Figure 4. The diameter of the circle thus formed is between 150 and 200 mm. The polystyrene support was chosen so as to not consider the backscattered radiation. We made sure beforehand that the bending radius shall not interfere with the measurements, and then the response of the PSF in these conditions was similar to the one observed in real use. The scintillating-fiber manufacturer gives the minimum bending diameter of 50 mm. The response versus bend radius was also evaluated by Hyer et al. and shows that the response of the dosemeter did not decrease significantly until there was a small radius of 40 mm (>5%) [27]. We have completed these data with a series of dose measurements in the sealed facility under different bending diameters from 250 to 100 mm and observed a deviation lower than 1% with an in-line use for bending diameter between 200 and 150 mm. This study will not be detailed here.

The calibration was performed at the LNHB with the RQT9 reference radiation quality for CT imaging with a 300 × 300 mm^2^ beam size and a source to detector distance of 120 cm. 

First, we measured the air kerma $K_{air}$ using the free-air ionization chamber and then placed the IVISCAN^®^ under the same irradiation conditions (distance, photon field, radiation quality, etc.). The calibration coefficient $N_{k,\mathrm{RQT}9}^{\mathrm{IVISCAN}}$ in air kerma per count of the IVISCAN^®^ dosemeter is written:

(1) $$N_{k,\mathrm{RQT}9}^{\mathrm{IVISCAN}}=\frac{K_{air}}{M_{\mathrm{RQT}9}}$$

where $M_{\mathrm{RQT}9}$ is the raw measurement of IVISCAN^®^ in counts for RQT9 reference radiation quality.

### 2.4. Repeatability

In order to get the measurement uncertainty associated with the X-ray source as low as possible, we performed these measurements under the following reference calibration conditions at LNHB. 

The source–detector distance was 100 cm and the beam area was 300 × 300 mm^2^. The measurements were performed over a period of 1000 ms compared to the time resolution of the IVISCAN^®^ dosemeter is 1 ms.

We obtained the measurement repeatability by performing a series of 10 measurements with the IVISCAN^®^ dosemeter for RQT9, RQR8, RQR9, and RQR10 radiation qualities. The measurement values are raw data in counts. Then, we evaluated the standard deviation σ and the expanded uncertainty $U\left(\bar{x}\right)$ with a coverage factor k=2, providing an approximate 95% confidence level such as:

(2) $$U\left(\bar{x}\right)=\frac{k\times \sigma }{\sqrt{n}},$$

where n is the number of measurements.

The measurements were performed at different dose rates depending on the radiation qualities. Patient entrance exposure rates of clinical CT units are typically lower than 60 mGy/s and around 1 mGy/s for a low-dose CT scan. Some specific and high-dose CT examinations gave exposure higher than 100 mGy/s (i.e., for middle ear or lumbar spine examination). The dose rates studied were between 0.55 and 1.56 mGy/s, close to the minimum dose rates used in CT examinations in order to evaluate the repeatability under least favorable clinical conditions.

### 2.5. Energy Dependence

#### 2.5.1. Theoretical Comparison

Dosemeters used for radiological exposures are calibrated to the energy of the X-ray spectrum as it would be for air-filled gas detectors such as ion chambers in air kerma. Although it is generally considered that scintillation efficiency is energy independent, this theory is not valid for low-energy beams as in diagnostic radiology. In this specific case, the X-ray energy-absorption properties of PSF are different from those of air and then the energy absorbed per unit mass within the scintillator volume is lower than the one in the same volume filled with air. As a result, the scintillation yield decreases relative to air kerma.

To study the energy dependence of the IVISCAN^®^ dosemeter, we first modeled the theoretical relative calibration coefficient $N_{k,Q}^{th}$ of the PSF in terms of air kerma for the radiation quality *Q*, based on the ratio of the mass-energy absorption coefficients ${\left[\frac{\mu_{en,Q}}{\rho }\right]}_{polystyrene}$ and ${\left[\frac{\mu_{tr,Q}}{\rho }\right]}_{air}$ of the PSF and air, respectively, for the same *Q* radiation quality. These coefficients are given by the NIST as a function of the energy of the X-rays [33]. Considering that the PSF is mainly composed of polystyrene, the corresponding $\frac{\mu_{en,Q}}{\rho }$ are those of polystyrene. The theoretical calibration coefficient $N_{k,Q}^{th}$ is given by:

(3) $$N_{k,Q}^{th}=\frac{{\left[\frac{\mu_{tr,Q}}{\rho }\right]}_{air}}{{\left[\frac{\mu_{en,Q}}{\rho }\right]}_{polystyrene}}$$

So we can deduce the relative theoretical calibration coefficient $N_{k,Q}^{th*}$ of the PSF normalized at RQT9 such that:

(4) $$N_{k,Q}^{th*}={\left[\frac{\mu_{tr,Q}}{\mu_{tr,\mathrm{RQT}9}}\right]}_{air}\times {\left[\frac{\mu_{en,\;\mathrm{RQT}9}}{\mu_{en,Q}}\right]}_{polystyrene}$$

A series of measurements was performed in N, RQR, RQT and CCRI radiation qualities in order to determine the corresponding $N_{k,Q}^{\mathrm{IVISCAN}}$ calibration coefficient for each radiation quality *Q* over the entire diagnostic energy range. It should be noted that each CT scan has its own radiation qualities, and they vary according to the examination protocols used, taking into account the voltage and filtration according to the protocols. The main CT manufacturers give half-value layers (HVLs) between 5 and 15 mm Al, which corresponds to effective energy $E_{eff}$ between 42 and 100 keV for voltages between 70 and 140 kVp.

The relative experimental calibration coefficient of the system $N_{k,Q}^{\mathrm{IVISCAN}*}$ is the IVISCAN^®^ calibration coefficient normalized at RQT9 and is given by:

(5) $$N_{k,Q}^{\mathrm{IVISCAN}*}=\frac{N_{k,Q}^{\mathrm{IVISCAN}}}{N_{k,\mathrm{RQT}9}^{\mathrm{IVISCAN}}}$$

Then, we compared the relative theoretical and experimental calibration coefficients of the dosemeter through the deviation $\Delta N_{k,Q}{}^{*}$ given by:

(6) $$\Delta N_{k,Q}{}^{*}=\frac{\left(N_{k,Q}^{\mathrm{IVISCAN}*}-N_{k,Q}^{th*}\right)}{N_{k,Q}^{th*}}\times 100$$

The set of radiation qualities used is represented in Table 1.

#### 2.5.2. Energy Correction Factor

This study was carried out with an IVISCAN^®^ solution installed on a Siemens SOMATOM^®^ Definition Edge CT scan (Siemens Healthiness GmbH, Erlangen, Germany).

According to the general formalism given in the IAEA international code of practice TRS-398 and following the calibration procedure, the air kerma in a medium-energy X-ray beam of quality *Q* is given by:

(7) $$K_{air,Q}=M_{Q}\times N_{k,\mathrm{RQT}9}^{\mathrm{IVISCAN}}\times k_{Q,\mathrm{RQT}9}$$

where $M_{Q}$ is the reading of the dosimeter IVISCAN^®^ in counts for a given radiation quality *Q*. We note here that $M_{Q}$ is independent of the temperature and pressure. $N_{k,\mathrm{RQT}9}^{\mathrm{IVISCAN}}$ is the calibration coefficient of IVISCAN^®^ dosimeter in RQT9 beam quality, as defined in Equation (1), and factor $k_{Q,\mathrm{RQT}9}$ is the dosemeter-specific factor which corrects for differences between the reference radiation quality RQT9 and the radiation quality *Q* being used.

In real use, the energy dependence correction factors $k_{Q,\mathrm{RQT}9}$ was defined during the installation process of IVISCAN^®^ on the CT scan through the HVL evaluation for each protocol and corresponding to the $N_{k,Q}^{\mathrm{IVISCAN}*}$ defined in Equation (5).

A series of n measurements were performed with IVISCAN^®^ and a pencil-type ionization chamber at 70, 80, 100, 120, and 140 kV and were compared. n=5 for the FLAT mode and n=3 for the Wedge mode. In this way, all CT scan voltage/filtration pairs were tested.

The $\Delta D_{Q}$ deviations between the dose measurement performed with IVIscan^®^ $D_{Q,\mathrm{IVISCAN}}$ and the dose measurement performed with the pencil ionization chamber $D_{Q,CI}$ is given by:

(8) $$\Delta D_{Q}=\frac{D_{Q,\mathrm{IVISCAN}}-D_{Q,CI}}{D_{Q,CI}}\times 100$$

where the $D_{Q,\mathrm{IVISCAN}}$ and $D_{Q,CI}$ correspond to the average doses of the n measurements.

### 2.6. Dose Rate Dependence

We obtained dose-rate dependence by irradiating a 1cm long PSF probe for 120 kVp X-ray beams and for dose rate between 2 mGy/s to more than 150 mGy/s in the sealed chamber. An additional series of measurement was carried out at the LNHB in RQT9 radiation quality and for dose rate between 5 μGy/s to 2 mGy/s in order to study the dependence for low dose rate with as much precision as possible. In the two cases, the dose rate variation was obtained by changing the tube current. Reference dose rate was obtained with X2 chamber and LNHB free-air primary chamber, respectively.

The validation measurements were then performed with an IVISCAN^®^ probe for CT tube current between 10 and 600 mA, corresponding to the minimum and the maximum value in clinical use and a tube voltage of 120 kVp. Reference dose rate was obtained with X2 chamber. The IVISCAN^®^ probe and the X2 chamber were placed on the CT couch, under the mattress, as in real-use, and stationary acquisition was launched for each current value. A step of 10 mA was applied for tube current between 10 and 100 mA, and a step of 100 mA was applied between 100 and 600 mA.

The deviation $\Delta \dot{D_{Q}}$ from the reference value is given by:

(9) $$\Delta \dot{D_{Q}}=\frac{\dot{D_{Q,\mathrm{IVISCAN}}}-\dot{D_{Q,ref}}}{\dot{D_{Q,ref}}}\times 100$$

where $\dot{D_{Q,\mathrm{IVISCAN}}}$ and $\dot{D_{Q,ref}}$ are, respectively, the dose rate for IVISCAN^®^ and the reference dosemeter for a given radiation quality *Q*.

### 2.7. Angular Dependence

Angular dependence measurements were performed by irradiating a 1 cm long PSF probe in a Siemens SOMATOM^®^ Definition Edge CT scan (Siemens Healthiness GmbH, Germany). The probe geometrical center was placed at the CT isocenter free-in-air, and the longitudinal axis of the probe was aligned along the Z-axis (X-ray tube rotation axis). Three stationary acquisitions for 120 kVp and 3 s per rotation were launched for reproducibility. Measurements were taken every 10 degrees from 0 to 360.

The deviation $\Delta \dot{D_{a}}$ for the angle from the mean value is represented by:

(10) $$\Delta \dot{D_{a}}=\frac{\dot{D_{a}}-\dot{D_{mean}}}{\dot{D_{mean}}}\times 100$$

where $\dot{D_{a}}$ is the dose rate for the angle a and $\dot{D_{mean}}$ the mean dose rate calculated for a complete rotation of 360°.

### 2.8. Stability with Cumulative Dose

PSF are widely used in particle physics where they are exposed to high levels of radiation. Hence, the degradation of their performance due to radiation damage has been extensively studied in the last two or three decades [34,35,36] This degradation is mainly caused by damages both to the plastic fiber base and to the scintillating molecules embedded in the base that result in transmission loss and reduction of scintillation efficiency, respectively. The overall effect is a loss of light and mainly depends on the absorbed dose and the type of radiation.

For this study, we irradiated an IVISCAN^®^ probe in the sealed chamber at 80 kV in 50 Gy irradiation steps up to 1000 Gy with a dose rate of 85 mGy/s. This setup refers to the one described in the IEC61674 standard and also allows us to verify that the system meets the performance requirements for use in radiodiagnostics. A series of 3 dose-rate measurements of 10 s each was performed between each irradiation step at 120 kVp and HVL of 8.6 mm Al. The dose rate values considered were obtained by calculating the average of the 3 measurements. The deviation $\Delta {\dot{D}}_{50Gy}$ as a percentage between each 50 Gy irradiation step is given by:

(11) $$\Delta {\dot{D}}_{50Gy}=\frac{{\dot{D}}_{n+1}-{\dot{D}}_{n}}{{\dot{D}}_{n}}\times 100$$

where ${\dot{D}}_{n}$ corresponds to the dose rate measured after the *n*th irradiation step.

### 2.9. Fiber Size Dependence

Here, we compared the response of two PSF probes of 0.5 mm diameter and 200 cm long for IVISCAN^®^ probe and 1 cm long for the second probe. The 1 cm PSF probe is made with the same scintillating material and clear optical fiber as the IVISCAN^®^ probe. The measurements were made under the different radiation qualities to cover the entire radiology energy range. The two probes were calibrated in RQT9 radiation quality at LNHB.

The deviation $\Delta D_{size}$ between the two probes response is given by:

(12) $$\Delta D_{size}=\frac{D_{1,PSF}-D_{\mathrm{IVISCAN}}}{D_{\mathrm{IVISCAN}}}\times 100$$

where $D_{1,PSF}$ and $D_{\mathrm{IVISCAN}}$ are the doses measured by the 1 cm PSF probe and the IVISCAN^®^ probe, respectively.

### 2.10. Homogeneity along the PSF

The homogeneity along the PSF was evaluated by irradiating an IVISCAN^®^ probe over a total length of 160 cm, limited by the maximum exploration range of the CT scan and measuring the resulting air kerma $D_{z,\mathrm{IVISCAN}}$. For this experiment, we used an IVISCAN^®^ dosemeter, which was installed on a Siemens SOMATOM^®^ Definition Edge CT scan (Siemens Healthiness GmbH, Germany). The measurements were performed for stationary acquisitions of 120 kV with the FLAT filter and a total beam collimation of 10 mm at 10 cm intervals in the z-axis direction. The reference air kerma $D_{ref}$ was obtained by irradiating an X2 chamber a its reference point in the same conditions. The deviation $\Delta D_{z}$ between the air kerma measured by IVISCAN^®^ and the reference is given by:

(13) $$\Delta D_{z}=\frac{D_{z,\mathrm{IVISCAN}}-D_{ref}}{D_{ref}}\times 100.$$

## 3. Results 

### 3.1. Repeatability

The results are presented in Table 2. The standard deviation is less than 0.039% and the expanded uncertainty of repeatability is between 0.017% and 0.025% depending on the radiation qualities and dose rates used (1.56 mGy/s and 0.55 mGy/s). The uncertainty decreases as the dose rate increases.

Given the range of dose rates found in CT imaging, this result demonstrates that the IVISCAN^®^ dosemeter has a very good measurement repeatability over the exposure range of interest in CT imaging.

### 3.2. Energy Dependence

#### 3.2.1. Theoretical Comparison

**Figure 5:**
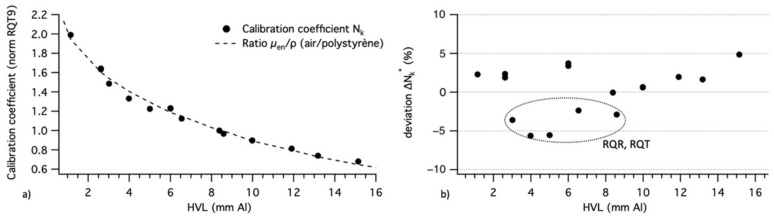
(**a**) Calibration coefficient $N_{k,Q}{}^{*}$ of IVISCAN^®^ (dots) and mass–energy-absorption coefficient ratio for air and polystyrene (line), normalized to RQT9 radiation quality for the entire diagnostic radiology energy range. (**b**) Deviation between IVISCAN^®^ and theoretical model for the entire diagnostic radiology energy range. Dotted circle shows the deviation for RQR and RQT radiation qualities.

The theoretical calibration coefficient curve and experimental calibration coefficients $N_{k,Q}{}^{*}$ are presented in Figure 5a. The data have been normalized to the RQT9 radiation quality. Error bars are not shown because they would be too small to be displayed.

Relative deviation $\Delta N_{k}{}^{*}$ (%) is presented in Figure 5b. The mean deviation is 2.7% with a maximum deviation of −5.5% for the RQR9 radiation quality, which correspond to the lower limit of CT effective energy.

We note that the mean deviation between the theoretical model fit curve and the theoretical values given by the NIST is 2.2% and the maximum deviation is 3.4%.

The N (Narrow) radiation quality’s energy spectrum resolution is smaller than those of the other radiation qualities, so the 1st HVL associated with the N spectra is fairly representative. Thus, the use of the 1st HVL in our graphs is less representative for RQT, RQR, and CCRI radiation qualities. We suggest that the larger $\Delta N_{k}{}^{*}$ for these radiation qualities comes from this consideration. We can therefore consider that there is a good agreement between the measurements and the theoretical values. Qualitatively, the response of the IVISCAN^®^ dosemeter is well defined by the ratios of the mass–energy absorption coefficients of air and polystyrene.

In quantitative terms, we observe a significant energy dependence from +22 to −32% for the air kerma measurement compared to the RQT9 reference radiation quality for HVL from 6 to 15 mm Al (42 to 100 keV), respectively. This energy dependence is due to the variability of the ratios of the mass–energy-absorption coefficients of PSF to air in this energy range and is fitted here by an exponential law. It is therefore necessary to compensate for this effect in order to give accurate air kerma values in CT imaging with PSF dosemeter technology.

The correction function was established by considering the values of $N_{k,Q}$ over several batches of IVISCAN^®^ dosemeters in order to take into account batch variability.

We note that the PSF energy dependence implies that one must have a good knowledge of the X-ray radiation quality to minimize the measurement errors under clinical use.

#### 3.2.2. Correction Factor

All the results presented below concern data automatically corrected by the IVIYOU^®^ software from the $k_{Q,\mathrm{RQT}9}$ factors.

Table 3 shows the deviation ΔD as a percentage between IVISCAN^®^ and the pencil-type ionization chamber for each voltage/filtration couple. Flat and Wedge correspond to the filter type label for the examination protocol.

For all radiation qualities, the mean deviation is always less than 1.84% in clinical use (70 to 140 kVp). It should be noted that the large majority of the examination protocols performed on this scanner are in Flat mode at 120 or 100 kV. In these two cases, the deviation is 0.77% and 1.02%, respectively.

Given these results, we can consider that the deviation from the reference value obtained after the correction is negligible.

We evaluated the energy dependence of the detector for X-rays photons from 20 to 140 kVp in order to cover the entire range of diagnostic radiology. The IVISCAN^®^ dosemeter has a significant energy dependence for the air kerma measurement requiring a correction in the CT range. This energy dependence is also manifest as a variation in sensitivity attributed to radiation quality changes, and the correction factor is directly correlated to the mass energy-absorption coefficients of polystyrene and air.

Once the dosemeter is calibrated in air kerma in the RQT9 radiation quality and after application of the correction factor, the deviation between IVISCAN^®^ and the reference is less than 1.84% regardless of the clinical use. For comparison purposes, according to the manufacturer, the X2 pencil ion chamber has an energy dependence of less than 5% for 70 to 150 kV, and generally, the same characteristics are observed for the other manufacturers.

### 3.3. Dose Rate Dependence

**Figure 6:**
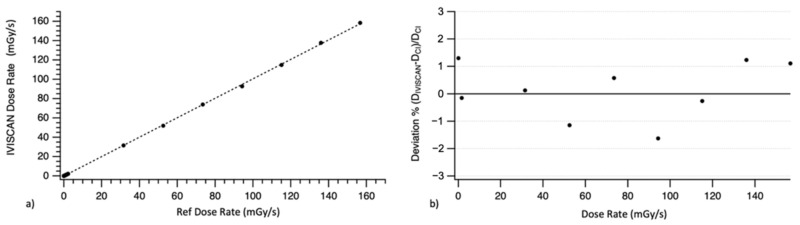
(**a**) IVISCAN^®^ response in air kerma rate related to reference ion chamber for air kerma rate between 0.005 and 160 mGy/s. (**b**) Deviation between air kerma rate measured by IVISCAN^®^ and reference ion chamber.

IVISCAN^®^ response was compared to reference air kerma rate measurement. The results obtained in the sealed chamber were presented in Figure 6a. The fitting curve is represented by a linear function $f\left(x\right)=ax+b$ with the intercept b=0 and the slope a=1.004 ±0.002. We also evaluated the deviation $\Delta \dot{D_{\mathrm{IVISCAN}}}$ between air kerma rate measured by IVISCAN^®^ and the reference ion chamber. The results presented in Figure 6b show that the maximum deviation is under ±2% each time.

**Figure 7:**
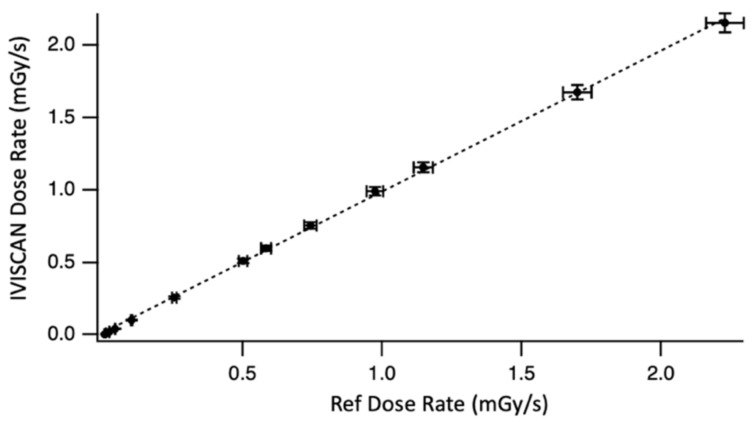
IVISCAN^®^ response related to LNHB air wall chamber WK07 for low dose rate between 0.005 and 2 mGy/s and RQT9 radiation quality.

Figure 7 shows the linearity for very low air kerma rates. High stability of LNHB beams and reference measurements allow us to perform these tests with a minimal uncertainty related to the environmental parameters, i.e., all the parameters other than those inherent to IVISCAN^®^. Here, the slope of the best-fit line is equal to 0.989±0.006.

**Figure 8:**
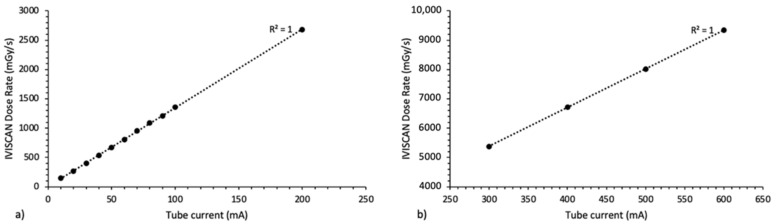
IVISCAN^®^ response in air kerma rate for the whole range of X-ray tube current for (**a**) small focal spot and (**b**) large focal spot, on Canon Aquilion ONE^TM^ Genesis.

The results obtained under clinical use on Aquilion ONE^TM^ Genesis were presented in Figure 8 for X-ray tube current from 10 to 600 mA and for (a) small focal spot and (b) large focal spot. As the air kerma rate is directly proportional to the tube current, the fitting curves are then represented by a linear function, and the R^2^ coefficient is equal to 1 for each one.

By comparison, technical specifications of the usual pencil ion chambers show a dose rate dependence between 2% and 5%. These results highlight the independence of the IVISCAN^®^ response in air kerma rate under the CT range and then the high response linearity of IVISCAN^®^ over the whole range of air kerma rates studied.

### 3.4. Angular Dependence

**Figure 9:**
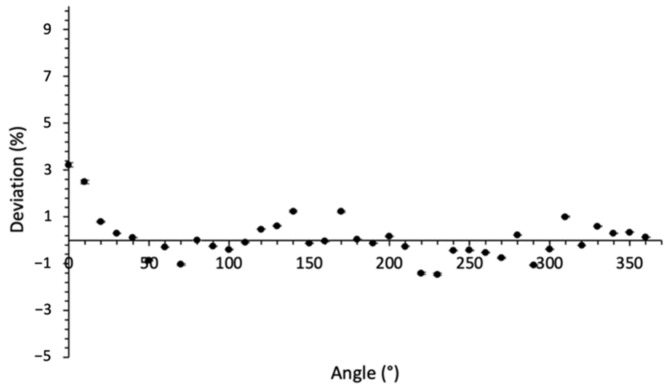
Deviation in % of the dose measured with a PSF from the average dose over 360°.

Figure 9 shows the deviation of the PSF response for each 10° irradiation angle from the mean value over 360°. The maximum deviation $\Delta \dot{D_{10}}$ of +2.5% was obtained for the first measurement and could be due to the instability of the CT beam at the beginning of the irradiation. We calculated a standard deviation of 0.8%, and the absolute uncertainty was 1.6% (with k = 2). Angular dependence results are within the recommendation limit of 3% (IEC61674) as for the pencil ion chambers.

Considering the uncertainty associated with the dose homogeneity delivered by the CT for a complete rotation of 360° of the X-ray tube and also to the probe position in the gantry, we can consider that the dosemeter response is independent of the CT beam angle.

### 3.5. Stability with Cumulative Dose

**Figure 10:**
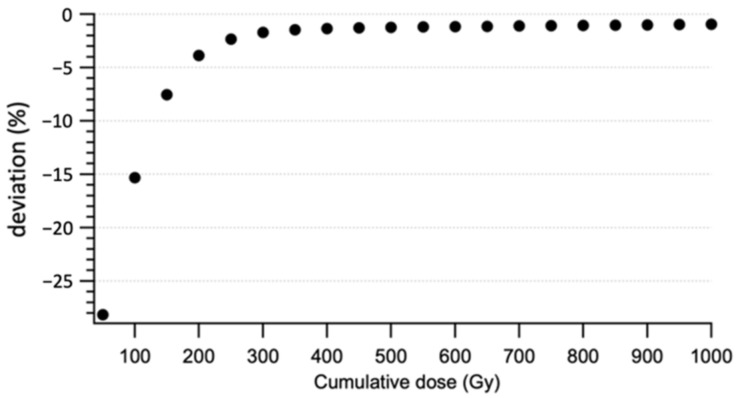
Deviation of IVISCAN^®^ dosemeter response as a function of cumulative dose. Dose rate deviations were evaluated for each 50 Gy step.

Figure 10 shows the variation $\Delta {\dot{D}}_{50Gy}$ between the dose rates measured for each 50 Gy step. This variation is exponential. Indeed, we observe a variation of about 25% of the measurement between 0 and 200 Gy of cumulative dose, and then the difference decreases rapidly under 1% of variation every 50 Gy from a cumulative dose of 500 Gy. According to IEC61674, the recommendation limit is 1% for a cumulative dose of 40 Gy.

These results obtained in the radiodiagnostic range supplement those of Carrasco et al. [37], who studied the radiation damage on the commercial PSF detector Exradin W1 in therapeutic beams. Indeed, they found that the rate of sensitivity loss decreased as the cumulative dose increased and radiation damage resulted in a 2.8% sensitivity loss under MV photon beam of 10 kGy [37].

This study shows that IVISCAN^®^ probe gain radiation resistance with increasing cumulative dose, and a minimal pre-irradiation of 500 Gy is required before the first use in order to have a satisfactory stable response according to the recommendation limit. Moreover, considering an average dose of a few mGy to a few tens of mGy delivered in CT and a three-yearly recurrence of dosemeter recalibration, the impact of the sensitivity loss can be disregarded.

### 3.6. Fiber Size Dependence

**Figure 11:**
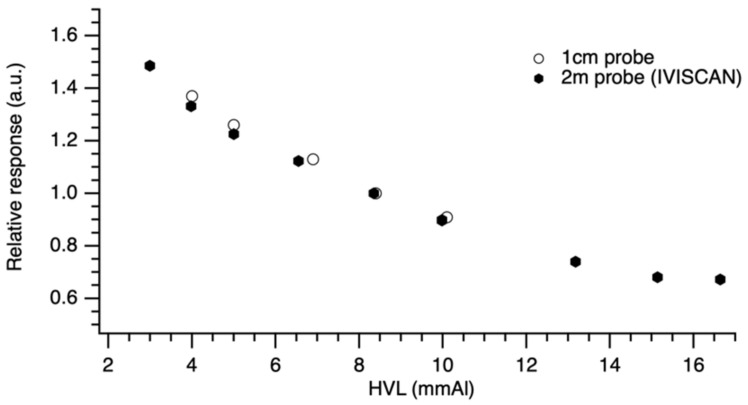
Relative response for 200 cm and 1 cm probe length under a wide range HVLs found in clinical routine. Data were normalized to RQT9 radiation quality.

The data presented in Figure 11 are normalized to the reference radiation quality RQT9 values in order to compare the PSF probe responses. As detailed in Table 4, we obtained a similar response with the two probe lengths with a maximum deviation $\Delta D_{size}$ < 2.7% between IVISCAN^®^ probe and 1 cm PSF probe. 

The calibration certificate indicates air kerma measurement uncertainties of 1.5% and 3.6% for IVISCAN^®^ probe and 1 cm probe, respectively (k = 2). On this basis, we can consider that the probe response is independent of the PSF length.

### 3.7. Homogeneity along the PSF

**Figure 12:**
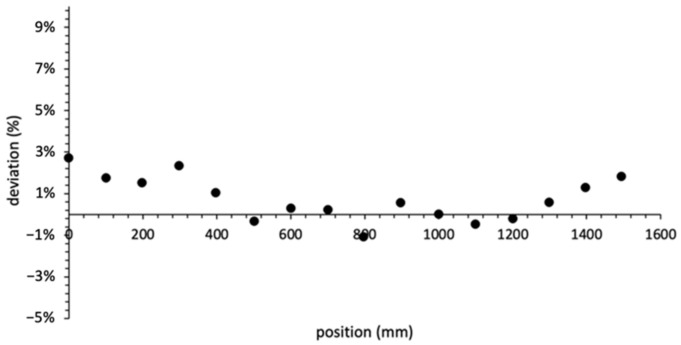
Relative deviation $\Delta D_{z}$ in % between the air kerma measured by IVISCAN^®^ and the reference for each z-axis irradiation position along the PSF; 1000 mm corresponds to the PSF center.

Figure 12 reports, for an IVISCAN^®^ solution, the homogeneity of the response along the PSF. The air kerma measurements were carried out using a thin collimation of 10 mm so as to minimize the irradiation position uncertainty. Position 0 corresponds to the PSF extremity on the head side of the CT couch. Considering the probe effective length of 2000 mm, the PSF center corresponds to the 1000 mm position on the graph.

Photon attenuation phenomena in optical fibers are widely known. We can mention a plurality of phenomena responsible for the total photon attenuation through the IVISCAN^®^ probe, and we categorize them in two groups: the fiber absorptions, characterized by the PSF and clear optical fiber attenuation length, and point losses due to both the splicings between PSF and clear optical fiber and the SMA connectors. These point losses constitute the largest part of the total attenuation in the probe. All these effects are corrected by IVIYOU^®^ software so as to minimize the response deviation along the probe. Comparison between IVISCAN^®^ dosemeter and reference measurements according to z-axis irradiation position shows a quite stable deviation $\Delta D_{z}$ between −1.1% and +2.7%. However, if we study in greater detail, we can observe that the deviation falls as we get closer to the PSF center. This can be explained by residual effects after software correction. These results highlight the good homogeneity of the response along the entire exploration area of the CT scan. Considering the specified rated length at 180 cm, the IVISCAN^®^ probe is fully compliant with the requirements for dosemeters in CT applications. By comparison, usual pencil ion chambers show a typically deviation of 3% along a 10 cm probe.

## 4. Discussion and Conclusions

In the present study, IVISCAN^®^ was evaluated in reference and clinical computed tomography radiation qualities in the French metrology laboratory LNHB and on CT scanners. We have shown that:

- Over the exposure range of interest, the IVISCAN^®^ dosemeter has an excellent measurement repeatability;
- The application of correcting factors on an air kerma RQT9 calibrated device allow us to overcome the probe dependence on low energies encountered in computed tomography clinical condition to achieve a deviation of less than 2% from the reference;
- The IVISCAN^®^ dosemeter is independent of air kerma rates, CT beam angle, and PSF length, and the response is homogeneous along the probe length;
- The IVISCAN^®^ dosemeter response is stable with cumulative dose after a pre-irradiation of 500 Gy.

These results demonstrate that the performance of the IVISCAN^®^ dosemeter meets metrology and dosimetry standards and is suitable in computed tomography applications and clinical conditions. Moreover, some environmental parameters such as pressure, temperature, and humidity have no bearing on the final result of the IVISCAN^®^ measurement, which reduce uncertainties comparing to pencil ion chambers.

As mentioned in the introduction, dosimetry in computed tomography is crucial, and the development of new validated dosimetry solutions in clinically radiation quality is an important step forward. With a millisecond time resolution and a high sensibility, the IVISCAN^®^ dosemeter also allows real-time dosimetry, which could increase radiovigilance with an early detection of machine problems or bad practice, leading to unnecessary dose excess and display real-time Local Diagnostic Reference Levels (LDRLs). In addition, having a personalized and accurate CT dosimetry for each patient is very important, especially for pediatric population and could be useful for epidemiological studies [10]. Moreover, with its detection length, the IVIscan device would allow us to perform quality control on large collimations much more easily than current methods, as well as on helical acquisitions. Altogether, it may therefore lead to a paradigm shift in this field since current CT reference dosimetric tools could not be used in clinical conditions [6].

## Figures and Tables

**Table 1:** LNHB radiation qualities used and corresponding tube voltage, HVL, and effective energy $E_{eff}$. * RQT9 is the reference radiation quality in CT imaging.

Radiation Quality	Tube Voltage (kVp)	HVL (mm Al)	$E_{eff}\;(\mathrm{keV})$
N30	30	1.15	23.32
N40	40	2.62	31.7
N60	60	6.00	46.0
N80	80	9.98	63.6
N100	100	13.18	82.5
N120	120	15.14	100.0
RQR6	80	3.01	33.53
RQR8	100	3.98	37.74
RQR9	120	5.00	41.97
RQR10	150	6.55	48.32
RQT9 *	120	8.38	56.21
CCRI135	135	8.59	57.0
CCRI180	180	11.9	74.5

**Table 2:** Standard deviation and absolute uncertainty (k = 2) for IVISCAN^®^ repeatability measurements for RQR8, RQR9, RQR10, and RQT9 radiation qualities at LNHB.

Radiation Quality	Standard Deviation σ (%)	Absolute Uncertainty U (%)	Air-Kerma Rate (mGy/s)
RQR8	0.034	0.022	0.86
RQR9	0.031	0.019	1.13
RQR10	0.027	0.017	1.56
RQT9	0.039	0.025	0.55

**Table 3:** Deviations ΔD in percent (%) between IVISCAN^®^ and the ionization chamber for two filter types, Flat and Wedge, and different tube voltages.

Filter	Voltage (kVp)				
	70	80	100	120	140
**Flat**	0.05	0.55	1.02	0.77	0.10
**Wedge**	1.17	1.79	1.84	1.53	0.57

**Table 4:** Response deviation between the 1cm and the 200 cm PSF length for different radiation qualities in diagnostic range.

Tube Voltage (kVp)	HVL (mm Al)	Deviation (%)
100	4	1.25
105	5	2.32
120	8.4	2.69
150	10	2.14

## Data Availability

Not applicable.

## References

[1] Lell M.M., Wildberger J.E., Alkadhi H., Damilakis J., Kachelriess M. (2015). Evolution in Computed Tomography: The Battle for Speed and Dose. Investig. Radiol..

[2] National Council on Radiation Protection and Measurements (2009). Ionizing Radiation Exposure of the Population of the United States.

[3] National Council on Radiation Protection and Measurements (2019). Medical Radiation Exposure of Patients in the United States.

[4] Institute of Radiation Protection and Nuclear Safety (2012). Exposure of the French Population to Ionizing Radiation Linked to Medical Diagnostic Procedures in 2012.

[5] Institute of Radiation Protection and Nuclear Safety (2019). Exposure of the French Population to Ionising Radiation—EXPRI Report IRSN.

[6] Damilakis J. (2021). CT Dosimetry: What Has Been Achieved and What Remains to Be Done. Investig. Radiol..

[7] Mathews J.D., Forsythe A.V., Brady Z., Butler M.W., Goergen S.K., Byrnes G.B., Giles G.G., Wallace A.B., Anderson P.R., Guiver T.A. (2013). Cancer risk in 680,000 people exposed to computed tomography scans in childhood or adolescence: Data linkage study of 11 million Australians. BMJ.

[8] De Gonzalez A.B., Salotti J.A., McHugh K., Little M.P., Harbron R.W., Lee C., Ntowe E., Braganza M.Z., Parker L., Rajaraman P. (2016). Relationship between paediatric CT scans and subsequent risk of leukaemia and brain tumours: Assessment of the impact of underlying conditions. Br. J. Cancer.

[9] Stewart F.A., Akleyev A.V., Hauer-Jensen M., Hendry J.H., Kleiman N.J., MacVittie T.J., Aleman B.M., Edgar A.B., Mabuchi K., Muirhead C.R. (2012). ICRP PUBLICATION 118: ICRP Statement on Tissue Reactions and Early and Late Effects of Radiation in Normal Tissues and Organs—Threshold Doses for Tissue Reactions in a Radiation Protection Context. Ann. ICRP.

[10] Till J.E., Beck H.L., Grogan H.A., Caffrey E.A. (2017). A review of dosimetry used in epidemiological studies considered to evaluate the linear no-threshold (LNT) dose-response model for radiation protection. Int. J. Radiat. Biol..

[11] National Academic Press Washington, Committee to Assess Health Risks from Exposure to Low Levels of Ionizing Radiation, National Research Council (2006). Health Risks from Exposure to Low Levels of Ionizing Radiation: BEIR VII—Phase 2.

[12] Rehani M.M., Yang K., Melick E.R., Heil J., Šalát D., Sensakovic W.F., Liu B. (2020). Patients undergoing recurrent CT scans: Assessing the magnitude. Eur. Radiol..

[13] Brambilla M., Vassileva J., Kuchcinska A., Rehani M. (2019). Multinational data on cumulative radiation exposure of patients from recurrent radiological procedures: Call for action. Eur. Radiol..

[14] Bastiani L., Paolicchi F., Faggioni L., Martinelli M., Gerasia R., Martini C., Cornacchione P., Ceccarelli M., Chiappino D., Della Latta D. (2021). Patient Perceptions and Knowledge of Ionizing Radiation from Medical Imaging. JAMA Netw. Open.

[15] International Commission on Radiological Protection (2000). Pregnancy and Medical Radiation. https://www.icrp.org/publication.asp?id=icrp%20publication%2084.

[16] Birks J.B., Firk F.W.K. (1965). The Theory and Practice of Scintillation Counting. Phys. Today.

[17] Borenstein S.R., Palmer R.B., Strand R.C. (1981). Optical Fibers and Avalanche Photodiodes for Scintillator Counters. Phys. Scr..

[18] Huston A., Justus B., Falkenstein P., Miller R., Ning H., Altemus R. (2001). Remote optical fiber dosimetry. Nucl. Instrum. Methods Phys. Res. Sect. B Beam Interact. Mater. At..

[19] O’Keeffe S., Fitzpatrick C., Lewis E., Al-Shamma’a A.I. (2008). A review of optical fibre radiation dosimeters. Sens. Rev..

[20] Lessard F., Archambault L., Plamondon M., Desprès P., Therriault-Proulx F., Beddar S., Beaulieu L. (2012). Validating plastic scintillation detectors for photon dosimetry in the radiologic energy Range: Validating Plastic Scintillation Detectors for Radiologic Energy Dosimetry. Med. Phys..

[21] Beaulieu L., Beddar S. (2016). Review of plastic and liquid scintillation dosimetry for photon, electron, and proton therapy. Phys. Med. Biol..

[22] Yoo W.J., Shin S.H., Jeon D., Hong S., Sim H.I., Kim S.G., Jang K.W., Cho S., Youn W.S., Lee B. (2014). Measurement of Entrance Surface Dose on an Anthropomorphic Thorax Phantom Using a Miniature Fiber-Optic Dosimeter. Sensors.

[23] Boivin J., Beddar S., Guillemette M.D., Beaulieu L. (2015). A novel tool for In Vivo dosimetry in diagnostic and interventional radiology using plastic scintillation detectors. Proceedings of the 7th WACBE World Congress on Bioengineering.

[24] Winslow J.F., Tien C., Hintenlang D.E. (2011). Organ dose and inherent uncertainty in helical CT dosimetry due to quasiperiodic dose distributions: Organ Dose and Uncertainty in Helical CT Dosimetry. Med. Phys..

[25] Hoerner M.R., Stepusin E.J., Hyer D., Hintenlang D.E. (2015). Characterizing energy dependence and count rate performance of a dual scintillator fiber-optic detector for computed tomography: Performance of a Dual Scintillator Fiber-Optic Detector. Med. Phys..

[26] Werneck M.M., Allil R.C. (2019). Plastic Optical Fiber Sensors: Science, Technology and Applications.

[27] Therriault-Proulx F., Beddar S., Beaulieu L. (2013). On the use of a single-fiber multipoint plastic scintillation detector for 192 Ir high-dose-rate brachytherapy: Single-Fiber Multipoint PSD for HDR Brachytherapy. Med. Phys..

[28] Hyer D.E., Fisher R.F., Hintenlang D.E. (2009). Characterization of a water-equivalent fiber-optic coupled dosimeter for use in diagnostic radiology. Med. Phys..

[29] Peralta L. (2018). Temperature dependence of plastic scintillators. Nucl. Instrum. Methods Phys. Res. Sect. A Accel. Spectrom. Detect. Assoc. Equip..

[30] Buranurak S., Andersen C., Beierholm A., Lindvold L. (2013). Temperature variations as a source of uncertainty in medical fiber-coupled organic plastic scintillator dosimetry. Radiat. Meas..

[31] Johnstone C., Therriault-Proulx F., Beaulieu L., Bazalova-Carter M. (2018). Characterization of a plastic scintillating detector for the Small Animal Radiation Research Platform (SARRP). Med. Phys..

[32] Gillet P., Munier M., Arbor N., Carbillet F., El Bitar Z. (2018). Evaluation of an optical scintillating fiber detector for CT dosimetry. Radiat. Meas..

[33] National Institute of Standards and Technology NIST Values of the Mass Attenuation Coefficient, μ/ρ, and the Mass Energy-Absorption Coefficient, Μen/ρ, as a Function of Photon Energy, for Compounds and Mixtures. https://physics.nist.gov/PhysRefData/XrayMassCoef/tab4.html.

[34] Aschenauer E.C., Baehr J., Gapienko V., Hoffmann B., Kharchilava A., Luedecke H., Nahnhauer R., Shanidze R. (1997). Development of Scintillating Fiber Detector Technology for High Rate Particle Tracking. arXiv.

[35] Kharzheev Y.N. (2019). Radiation Hardness of Scintillation Detectors Based on Organic Plastic Scintillators and Optical Fibers. Phys. Part. Nucl..

[36] Beddar S., Beaulieu L. (2016). Scintillation Dosimetry.

[37] Carrasco P., Jornet N., Jordi O., Lizondo M., Latorre-Musoll A., Eudaldo T., Ruiz A., Ribas M. (2014). Characterization of the Exradin W1 scintillator for use in radiotherapy. Med. Phys..

